# Male View on Aesthetic Procedures: A Local Survey

**DOI:** 10.7759/cureus.75939

**Published:** 2024-12-18

**Authors:** Yosra F Buhiliga, Hussam F Alkhars, Maha Alqahtani, Abdullah AlAlwan, Ali F Alkhars

**Affiliations:** 1 Plastic and Reconstructive Surgery, Dammam Medical Complex, Dammam, SAU; 2 Surgery, King Faisal University, Al Hofuf, SAU; 3 Surgery, College of Medicine, King Saud University, Riyadh, SAU; 4 Plastic and Reconstructive Surgery, King Faisal University, Al Ahsa, SAU; 5 Medical School, King Faisal University, Al Ahsa, SAU

**Keywords:** aesthetic procedures, attitude, awareness, male, perception, prevalence, saudi arabia

## Abstract

Background

Cosmetic procedures have become increasingly popular worldwide; however, male participation remains relatively low, especially in conservative societies like Saudi Arabia. This study explores the awareness, attitudes, and experiences of men concerning aesthetic procedures in Saudi Arabia, with a focus on sociodemographic factors and sources of information. This study aimed to assess male awareness, attitudes, and experiences with aesthetic procedures in Saudi Arabia and identify factors influencing their engagement with these interventions.

Methodology

A cross-sectional study was conducted involving 818 male participants from various regions in Saudi Arabia. Data on sociodemographic profiles, awareness levels, and attitudes toward cosmetic procedures were collected using a structured questionnaire. Descriptive statistics, chi-square tests, and logistic regression models were used for data analysis performed using SPSS version 26 (IBM Corp., Armonk, NY, USA).

Results

The study found that 504 (61.6%) participants had never undergone a cosmetic procedure. The most common reasons for not undergoing such procedures included the social image of men (198, 39.2%), cost (145, 28.7%), and no health issues requiring aesthetic intervention (66, 13.1%). Awareness of cosmetic procedures was relatively low, with 223 (27.3%) participants rating their awareness as “low” and 101 (12.3%) as “very low.” Social media (333, 40.7%), physicians (211, 25.8%), and television (144, 17.6%) were the primary sources of information. Significant factors influencing attitudes included age, education level, employment status, and marital status, all of which had a significant p-value below 0.05.

Conclusions

Awareness and acceptance of cosmetic procedures among Saudi males are increasing, influenced by various cultural, societal, and personal factors. This study highlights the need for targeted campaigns and education to promote understanding and acceptance. Further research is needed to examine how these attitudes evolve and their implications for the cosmetic industry and healthcare providers.

## Introduction

Plastic and reconstructive surgery and aesthetic surgery are related branches of medicine with the primary aim of improving the appearance and restoring the function of any part of the body. To classify them further, plastic surgery focuses on repairing and reconstructing medical defects, while aesthetic surgery focuses on enhancing the appearance of a healthy individual [1].

Aesthetic procedures, in general, are known to be dominated and linked with the female gender due to the societal standards of what beauty and the appearance of an individual should be. Studies have found a higher percentage of 90% in both cosmetic procedures performed and knowledge about them among females compared to males [2-4]. Recent data provided by the American and German associations of aesthetic plastic surgery showed an increasing number of men accepting and undergoing different aesthetic procedures [5-8]. However, there is still a lack of information on the prevalence, acceptance, motivations, and obstacles men face regarding aesthetic procedures [9].

This study aims to navigate the male view regarding different aesthetic procedures by exploring the motivations, societal influences, and implications of gender dynamics. This study would help deepen the understanding of how masculinity responds to the increasing popularity of aesthetic procedures.

## Materials and methods

In this cross-sectional study conducted from May to November 2024 in Saudi Arabia, we included male participants of Saudi nationality aged 18 years and above and living in Saudi Arabia. We excluded non-Saudi participants, those not living in Saudi Arabia, those younger than 18 years of age, and the female gender. We used a non-probability convenience sampling to invite male participants who met the inclusion and exclusion criteria. We distributed an online Google Forms questionnaire via social media platforms, shopping malls, and hospitals. The questionnaire was written in Arabic and divided into four sections. The first section focused on demographic data, including region, age, education level, employment status, monthly income, and marital status. In the second section concerning awareness and knowledge of aesthetic procedures, we assessed the participants’ general knowledge in a single question in which they, in their opinion, would comment on the level of their knowledge. We also enquired about the sources that provided the information to them. In the third section concerning the history of undergoing aesthetic procedures and reasons for not doing any, we assessed whether the participant had undergone an aesthetic procedure for either cosmetic or medical-related reasons. We also assessed the reasons and obstacles of not undergoing an aesthetic procedure. In the fourth section concerning attitudes and perception toward aesthetic procedures, we included factors that would influence an individual to undergo an aesthetic procedure and assessed the participant’s perception toward them, as well as the general acceptance of the aesthetic procedures. Subscale scores were computed by taking the mean value for items associated with each subscale. The total score, referred to as acceptance, was computed by calculating the mean across all items.

The questionnaire was designed in Arabic language and was verified by a language expert. The Lawshe method was used to determine the validity of the questionnaire. Seven experts in the field were consulted about the questionnaire and verified the content of the questionnaire. The content validity ratio was calculated accordingly. Questions with a content validity ratio below 0.99 were eliminated from the questionnaire. The reliability and validity of the questionnaire were assessed via a pilot study that involved 122 participants. The data collected in the pilot study was not included in the final analysis.

First, we gathered the questionnaire data and collected them using the Google Forms of the questionnaire that we published. Second, data was grouped into a Microsoft Excel sheet (Microsoft Corp., Redmond, WA, USA). Then, we translated the questionnaire into the English language and verified it by a language expert (see Appendices for the English version of the questionnaire). The statistical analysis was conducted using SPSS version 26 (IBM Corp., Armonk, NY, USA). Descriptive statistics were employed to summarize the sociodemographic profiles, awareness, and experiences of the male participants regarding aesthetic procedures. Chi-square tests were conducted to examine associations between categorical variables such as region, age, education, employment status, income, marital status, awareness levels, and attitudes toward cosmetic procedures, with p-values <0.05 determining statistical significance.

Data collection was gathered carefully respecting the privacy of each participant during each step of the study. Ethical approval was obtained from the Research Ethics Committee at King Faisal University (reference code: KFU-REC-2024-NOV-ETHICS2843).

## Results

Table 1 shows the sociodemographic profile of 818 male participants from Saudi Arabia. The study sample was nearly equally distributed across different regions, with the Northern (216, 26.4%) and Central (202, 24.7%) regions having the highest representation, followed by the Western (193, 23.6%), Southern (104, 12.7%), and Eastern (103, 12.6%) regions. Age ranged from 19 to more than 55 years, with a mean age of 39.5 ± 11.9 years. Regarding education, a large proportion of participants had attained higher education, with 363 (44.4%) having a postgraduate degree, 152 (18.6%) holding a university degree, and 149 (18.2%) having a diploma. The remaining participants had either basic education (75, 9.2%) or no formal education (79, 9.7%). Employment status revealed that more than half of the participants (421, 51.5%) were employed, while 200 (24.4%) were not working, and 197 (24.1%) were students. Monthly income varied, with the largest group reporting 10,000-20,000 SR (280, 34.2%), followed by those earning 5,000-10,000 SR (269, 32.9%). Smaller groups earned less than 5,000 SR (141, 17.2%) or more than 20,000 SR (128, 15.6%). Regarding marital status, most participants were married (395, 48.3%), followed by single individuals (273, 33.4%), and those who were divorced or widowed (150, 18.3%).

**Table 1:** Sociodemographic characteristics of study participants (n = 818). The data were represented as (N) and (%) for participants in each question.

Sociodemographics	N	%
Region		
Central Region	202	24.7%
Northern Region	216	26.4%
Eastern Region	103	12.6%
Western Region	193	23.6%
Southern Region	104	12.7%
Age in years		
19–25	182	22.2%
26–35	218	26.7%
36–45	193	23.6%
46–55	127	15.5%
>55	98	12.0%
Education level		
Not educated	79	9.7%
Basic education	75	9.2%
Diploma	149	18.2%
University education	152	18.6%
Postgraduate	363	44.4%
Employment status		
Not working	200	24.4%
Student	197	24.1%
Employee	421	51.5%
Monthly income		
<5,000 SR	141	17.2%
5,000–10,000 SR	269	32.9%
10,000–20,000 SR	280	34.2%
>20,000 SR	128	15.6%
Marital status		
Single	273	33.4%
Married	395	48.3%
Divorced/Widow	150	18.3%

Regarding male awareness of aesthetic procedures (Table 2), most males appeared to have a limited understanding. Specifically, 223 (27.3%) rated their awareness as “low,” while 216 (26.4%) classified it as “moderate.” Only 185 (22.6%) considered themselves “highly aware,” and 93 (11.4%) reported having “very high” awareness. Additionally, 101 (12.3%) indicated that their awareness was “very low.” Regarding sources of information, the majority relied on social media (333, 40.7%), followed by physicians (211, 25.8%) and television (144, 17.6%). Friends and family were cited less frequently at 97 (11.9%), while other sources were cited by 33 (4.0%) participants.

**Table 2:** Awareness and knowledge of male participants about aesthetic procedures (n = 818). The data are presented as N and % for participants on each question.

Awareness	N	%
What is your awareness level of aesthetic procedures?		
Very low	101	12.3%
Low	223	27.3%
Moderate	216	26.4%
High	185	22.6%
Very high	93	11.4%
Source of your information about aesthetic procedures?		
Physicians	211	25.8%
Social media	333	40.7%
TV	144	17.6%
Friends/Relatives	97	11.9%
Others	33	4.0%

**Figure 1:**
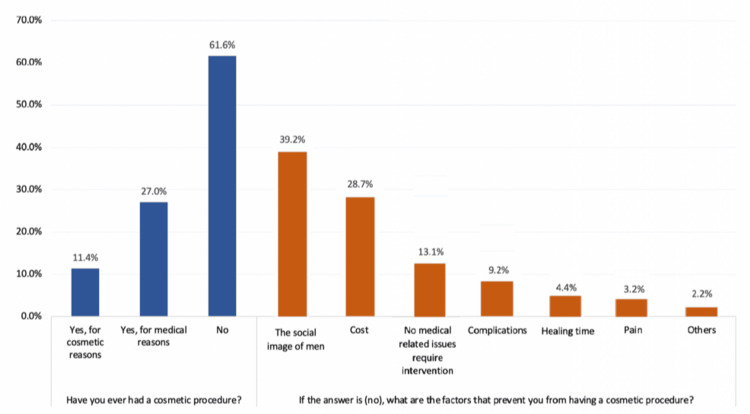
Male participants’ history of undergoing aesthetic procedures and reasons for not doing them. The data are presented as % for participants on each question.

Figure 1 provides an overview of participants’ experiences with cosmetic procedures. A majority of 504 (61.6%) reported never having undergone a cosmetic procedure, while 93 (11.4%) had undergone procedures for cosmetic reasons, and 221 (27.0%) for medical reasons. Among those who had not undergone such procedures, the most common reasons were the social image of men (198, 39.2%), cost (145, 28.7%), and no health problems requiring cosmetic intervention (66, 13.1%). Other reasons included complications (46, 9.2%), healing time (22, 4.4%), pain (16, 3.2%), and other factors (11, 2.2%).

Male attitudes and perceptions regarding cosmetic procedures are summarized in Table 3. In total, 248 (30.3%) participants agreed that cosmetic procedures are primarily aimed at improving one’s external appearance, with an additional 157 (19.2%) strongly agreeing. Conversely, 102 (12.5%) disagreed, and 95 (11.6%) strongly disagreed. When asked if societal standards of beauty influence cosmetic procedures, 216 (26.4%) agreed, and 297 (36.3%) strongly agreed. However, nearly 73 (8.9%) disagreed, 44 (5.4%) strongly disagreed, and 188 (23.0%) remained neutral. Participants were also asked about whether cosmetic procedures are undertaken due to pressure from friends or relatives. While 209 (25.6%) disagreed, 97 (11.9%) strongly disagreed, 195 (23.8%) remained neutral, 216 (26.4%) agreed, and 101 (12.3%) strongly agreed. Regarding job-related motivations for cosmetic procedures, 186 (22.7%) strongly agreed, 297 (36.3%) agreed, 117 (14.3%) disagreed, 102 (12.5%) strongly disagreed, and 116 (14.2%) remained neutral. When assessing health-related motivations, about 208 (25.4%) participants disagreed, and 201 (24.6%) agreed that health issues drive individuals to seek these procedures, with a notable 216 (26.4%) remaining neutral. In the assessment of whether individuals undergo cosmetic procedures for improving self-confidence, 205 (25.1%) agreed, and 101 (12.3%) strongly agreed. On the other hand, 215 (26.3%) disagreed, and 107 (13.1%) strongly disagreed, with the rest remaining neutral (190, 23.2%). On assessing the general opinion on aesthetic procedures, nearly half of the participants disagreed (407, 49.8%).

**Table 3:** Male participants’ attitudes and perceptions toward aesthetic procedures. The data are represented as N and % for participants on each question.

Attitude	Strongly disagree		Disagree		Neutral		Agree		Strongly agree	
	N	%	N	%	N	%	N	%	N	%
The cosmetic procedure is performed to improve a person’s external appearance	95	11.6%	102	12.5%	226	27.6%	248	30.3%	157	19.2%
The cosmetic procedure is done because of society’s standard of beauty	44	5.4%	73	8.9%	188	23.0%	216	26.4%	297	36.3%
The cosmetic procedure is performed due to pressure from relatives and friends	97	11.9%	209	25.6%	195	23.8%	216	26.4%	101	12.3%
The cosmetic procedure is done as a means to obtain a job or job requirements	102	12.5%	117	14.3%	116	14.2%	297	36.3%	186	22.7%
The cosmetic procedure is performed due to health problems	96	11.7%	208	25.4%	216	26.4%	201	24.6%	97	11.9%
Cosmetic procedures are performed to increase self-confidence	107	13.1%	215	26.3%	190	23.2%	205	25.1%	101	12.3%
General opinion on aesthetic procedures	107	13.1%	407	49.8%	102	12.5%	89	10.9%	113	13.8%

Table 4 shows factors associated with male awareness of aesthetic procedures in Saudi Arabia. Statistically significant differences were found with age and marital status (p < 0.05). Awareness levels varied across regions (p = 0.478), with 74 (36.6%) participants in the Central Region reporting high awareness compared to 64 (29.6%) in the Northern Region. Similarly, age significantly influenced awareness, with 87 (40.0%) participants aged 26-35 years reporting high awareness (p = 0.00001). Education level showed no significant impact, with high awareness ranging from 23 (30.7%) among those with basic education to 55 (36.9%) among diploma holders (p = 0.725). Similarly, employment status and monthly income had no significant associations, as high awareness ranged from 141 (33.5%) among employees to 53 (37.6%) among participants earning less than 5,000 SR. Marital status showed a significant association, with 153 (33.7%) married participants reporting high awareness (p = 0.0003). Lastly, prior experience with cosmetic procedures did not significantly affect awareness (p = 0.259), as 35 (37.6%) of those who had undergone procedures for cosmetic reasons had high awareness compared to 175 (34.7%) of those who had never undergone a procedure.

**Table 4:** Factors associated with male awareness of aesthetic procedures. All statistical methods used were two-tailed with an alpha level of 0.05 considering significance (*) if the p-value is less than or equal to 0.05.

Factors	What is your awareness level of aesthetic procedures?						P-value
	low		Moderate		High		
	N	%	N	%	N	%	
Region							0.478
Central Region	68	33.7%	60	29.7%	74	36.6%	
Northern Region	97	44.9%	55	25.5%	64	29.6%	
Eastern Region	40	38.8%	30	29.1%	33	32.0%	
Western Region	79	40.9%	45	23.3%	69	35.8%	
Southern Region	40	38.5%	26	25.0%	38	36.5%	
Age in years							0.00001*
19–25	82	45.1%	41	22.5%	59	32.4%	
26–35	53	24.3%	78	35.8%	87	40.0%	
36–45	91	47.6%	54	28.0%	48	24.9%	
46–55	57	44.9%	43	33.6%	27	21.6%	
>55	36	36.7%	23	23.5%	39	39.8%	
Education level							0.725
Not educated	26	32.9%	25	31.6%	28	35.4%	
Basic education	34	45.3%	18	24.0%	23	30.7%	
Diploma	57	38.3%	37	24.8%	55	36.9%	
University education	65	42.8%	34	22.4%	53	34.9%	
Postgraduate	142	39.1%	102	28.1%	119	32.8%	
Employment status							0.876
Not working	82	41.0%	47	23.5%	71	35.5%	
Student	76	38.6%	55	27.9%	66	33.5%	
Employee	166	39.4%	114	27.1%	141	33.5%	
Monthly income							0.448
<5,000 SR	54	38.3%	34	24.1%	53	37.6%	
5,000–10,000 SR	113	42.0%	63	23.4%	93	34.6%	
10,000–20,000 SR	114	40.7%	80	28.6%	86	30.7%	
>20,000 SR	43	33.6%	39	30.5%	46	35.9%	
Marital status							0.0003*
Single	120	44.0%	64	23.4%	89	32.6%	
Married	109	38.7%	133	27.7%	153	33.7%	
Divorced/Widow	51	34.0%	56	37.3%	43	28.7%	
Have you ever had a cosmetic procedure?							0.259
Yes, for cosmetic reasons	28	30.1%	30	32.3%	35	37.6%	
Yes, for medical reasons	95	43.0%	58	26.2%	68	30.8%	
No	201	39.9%	128	25.4%	175	34.7%	

Table 5 assesses the factors influencing male attitudes toward aesthetic procedures in Saudi Arabia. The region, age, education, employment status, income, previous cosmetic procedures, and awareness levels did not show significant differences in attitudes, with significant p-values in age, education level, employment, and marital status. Age between 26 and 35 years showed a positive attitude of 28.0% (p = 0.0006). For the education level, postgraduates showed the most positive attitude at 30.0% compared to the others with a p-value of 0.00001. Employment status showed that employees had the highest positivity among the others at 27.6% with a p-value of 0.00001. Marital status showed a significant association (p = 0.00001). Specifically, married individuals were less likely to have negative attitudes (37.5%) compared to single individuals (13.6%).

**Table 5:** Factors associated with male attitudes and perceptions of aesthetic procedures. All statistical methods used were two-tailed with an alpha level of 0.05 considering significance (*) if the p-value is less than or equal to 0.05.

Factors	Males attitude toward aesthetic procedures						P-value
	Negative		Neutral		Positive		
	N	%	N	%	N	%	
Region							0.912
Central Region	130	64.4%	42	20.8%	30	14.9%	
Northern Region	136	63.0%	47	21.8%	33	15.3%	
Eastern Region	63	61.2%	25	24.3%	15	14.6%	
Western Region	121	62.7%	49	25.4%	23	11.9%	
Southern Region	64	61.5%	28	26.9%	12	11.5%	
Age in years							0.0006*
19–25	112	61.5%	43	23.6%	27	14.8%	
26–35	104	47.7%	53	24.3%	61	28.0%	
36–45	126	65.3%	32	16.6%	35	18.1%	
46–55	76	59.8%	34	26.8%	17	13.4%	
>55	66	67.3%	19	19.4%	13	13.3%	
Education level							0.00001*
Not educated	42	53.2%	26	32.9%	11	13.9%	
Basic education	48	64.0%	15	20.0%	12	16.0%	
Diploma	90	60.4%	38	25.5%	21	14.1%	
University education	104	68.4%	28	18.4%	20	13.2%	
Postgraduate	170	46.8%	84	23.1%	109	30.0%	
Employment status							0.00001*
Not working	122	61.0%	49	24.5%	29	14.5%	
Student	131	66.5%	48	24.4%	18	9.1%	
Employee	211	50.1%	94	22.3%	116	27.6%	
Monthly income							0.235
<5,000 SR	89	63.1%	33	23.4%	19	13.5%	
5,000–10,000 SR	158	58.7%	65	24.2%	46	17.1%	
10,000–20,000 SR	187	66.8%	66	23.6%	27	9.6%	
>20,000 SR	80	62.5%	27	21.1%	21	16.4%	
Marital status							0.00001*
Single	172	63.0%	64	23.4%	37	13.6%	
Married	160	40.5%	87	22.0%	148	37.5%	
Divorced/Widow	82	54.7%	40	26.7%	28	18.7%	
Have you ever had a cosmetic procedure?							0.793
Yes, for cosmetic reasons	57	61.3%	23	24.7%	13	14.0%	
Yes, for medical reasons	137	62.0%	57	25.8%	27	12.2%	
No	320	63.5%	111	22.0%	73	14.5%	
What is your awareness level of aesthetic procedures?							0.863
low	203	62.7%	77	23.8%	44	13.6%	
Moderate	132	61.1%	55	25.5%	29	13.4%	
High	179	64.4%	59	21.2%	40	14.4%	

## Discussion

This study aimed to explore and assess the awareness, attitudes, and factors influencing Saudi males’ perceptions and acceptance of aesthetic procedures. The study also sought to explore and highlight beneficial information and demographic trends related to the uptake of these treatments. The study findings showed that most male participants in Saudi Arabia have never undergone a cosmetic procedure, which aligns with findings from international studies reporting a lower prevalence of cosmetic procedures among men compared to women [10]. However, about one-fourth of the men surveyed had undergone procedures for medical reasons, reflecting a growing acceptance of cosmetic interventions for health-related issues [11]. The primary reasons for not undergoing procedures were concerns about the social image of men, cost, and no medical issues requiring an intervention, which is consistent with global trends where financial considerations and social perceptions play significant roles in decision-making [11-13]. A systematic review conducted by Cohen et al. [14] revealed a lower incidence of aesthetic procedures among males compared to females, mainly for cosmetic reasons. On the other hand, according to studies, the incidence of aesthetic procedures among males has been steadily increasing [15]. According to the International Society of Aesthetic Plastic Surgery (ISAPS), men accounted for roughly 14.3% of all cosmetic surgical procedures worldwide in 2023, up from 13.5% in 2018 [16]. This rise reflects a growing acceptance and interest in aesthetic procedures among men [17]. In the United States, the American Society of Plastic Surgeons (ASPS) reported that men underwent 289,360 aesthetic surgical procedures and 820,123 non-surgical procedures in 2020 [18]. The most common procedures for men include rhinoplasty, eyelid surgery, and liposuction [15,18]. Similarly, the incidence of aesthetic procedures among males in Saudi Arabia is on the rise [19]. A study by Alharethy found that common procedures include rhinoplasty, eyelid surgery, and liposuction [20]. Typically, these men were university graduates, married, employed, and aged 20 to 40 years, reflecting a growing acceptance of aesthetic procedures similar to global trends.

Our study on male awareness of aesthetic procedures in Saudi Arabia indicated that many men have a limited understanding of these treatments. Alshehri et al. found that only 22.6% of Saudi men consider themselves “highly aware” of aesthetic procedures, while 27.3% rate their awareness as “low,” which is consistent with our findings. This limited awareness is concerning, given the growing global trend of men seeking cosmetic treatments. This is in contrast to findings from a previous study from Saudi Arabia where social media and digital platforms significantly boosted awareness of cosmetic procedures [21]. Research conducted in the United States found that social media plays a critical role in influencing men’s decisions to seek cosmetic treatments [22]. The current study’s findings align with those of a previous study, where social media was at 40.7%, followed by physicians at 25.8%, and television at 17.6%. These results highlight the need for targeted awareness campaigns and educational initiatives aimed at enhancing understanding and acceptance of aesthetic procedures among Saudi men. A study conducted in Majmaah, Saudi Arabia, found that social media is the most common source of information about cosmetic procedures among males, followed by physicians and television [11]. This finding aligns with international research that indicates digital platforms significantly contribute to raising awareness about cosmetic procedures.

Considering male attitudes toward aesthetic procedures, the study examined the varied attitudes of Saudi males toward cosmetic procedures. While many view these treatments as a way to enhance their appearance, many disagree, reflecting a range of opinions. This variation suggests that not all men see aesthetic enhancements as essential, indicating cultural resistance to altering one’s appearance for cosmetic reasons. Considering societal beauty standards, many men recognize their influence, while some remain neutral or disagree. This suggests that male beauty standards are still evolving and less established than those for women. Additionally, the effect of social factors, such as family and friends, appears to be not significant for men, indicating less societal pressure to chase cosmetic procedures. Even though responses regarding job-related motivations were mixed, a large proportion agreed that cosmetic procedures could improve their professional appearance, while some remained neutral or disagreed. This suggests that professional appearance is a significant factor for most men when considering cosmetic treatments. Similarly, health-related motivations for cosmetic procedures are not widely supported, with most men viewing these treatments as aesthetic rather than necessary for health.

Studies have shown that male attitudes toward cosmetic procedures are becoming increasingly accepting, although cultural and societal factors still play a significant role. A study by Abbas and Karadavut [23] highlighted that body image dissatisfaction and media exposure significantly influence men’s consideration of cosmetic surgery. In Saudi Arabia, a study by Al Hindi et al. [11] showed that younger, educated men are increasingly accepting of cosmetic procedures. This trend is partly motivated by the desire to enhance their appearance and gain professional advantages. However, traditional views and concerns about how society perceives these choices can still create obstacles. Overall, these findings indicate a changing landscape in which male acceptance of cosmetic procedures is growing, although motivations are complex and cultural influences remain significant.

This study stands out as it explores specifically Saudi males’ perceptions, attitudes, and awareness toward aesthetic procedures without focusing on females, as done in previous studies. The study included participants from different regions of Saudi Arabia. However, certain limitations should be acknowledged. First, distributing the online questionnaire via social media platforms, shopping malls, and hospitals could have shown a bias toward community members who did not get an opportunity to access the questionnaire. Second, the sample size was not distributed equally throughout all regions of Saudi Arabia. As a result, the findings of the study may not represent the entire population of Saudi Arabia.

## Conclusions

This study revealed that male participants in Saudi Arabia have limited awareness of aesthetic procedures, with most reporting low-to-moderate levels of understanding. A significant proportion of men had not undergone cosmetic treatments, citing factors such as societal views of masculinity, cost, and non-existing medical issues. The primary sources of information about these procedures were social media, physicians, and television. While various factors influence attitudes toward aesthetic procedures, societal expectations and job-related pressures are universally seen as the main drivers. Age, education level, employment status, and marital status also play a role. Overall, growing awareness and acceptance of cosmetic procedures among Saudi men are influenced by cultural, societal, and personal factors. Some embrace these treatments for confidence, while others resist due to cultural norms. As societal views evolve, further research is needed to understand how these attitudes change and their implications for the cosmetic industry in Saudi Arabia.

## Appendices

**Table 6:** Questionnaire on male views on aesthetic procedures. The questionnaire is set in four sections in a multiple-choice answer format. Each participant would choose their answer from sociodemographics to their own opinions on aesthetic procedures.

Section one: sociodemographic data	
Region	
Central Region	
Northern Region	
Eastern Region	
Western Region	
Southern Region	
Age in years	
19–25	
26–35	
36–45	
46–55	
>55	
Education level	
Not educated	
Basic education	
Diploma	
University education	
Postgraduate	
Employment status	
Not working	
Student	
Employee	
Monthly income	
<5,000 SR	
5,000–10,000 SR	
10,000–20,000 SR	
>20,000 SR	
Marital status	
Single	
Married	
Divorced / widow	
Section two: awareness and knowledge of male participants about aesthetic procedures	
What is your awareness level of aesthetic procedures?	
Very low	
Low	
Moderate	
High	
Very high	
Source of your information about aesthetic procedures?	
Physicians	
Social media	
TV	
Friends/Relatives	
Others	
Section three: history of undergoing aesthetic procedures and reasons for not undergoing procedures	
Have you ever done an aesthetic procedure?	
Yes, for cosmetic reasons	
Yes, for medical reasons	
No	
If the answer was (no), which of the following reasons was the reason for not undergoing them?	
The social image of men	
Cost	
No medical-related issues require aesthetic intervention	
Complications	
Pain	
Healing time	
Others	
Section four: attitudes and perceptions toward aesthetic procedures	
The cosmetic procedure is performed to improve a person’s external appearance	
Strongly agree	
Agree	
Neutral	
Disagree	
Strongly disagree	
The cosmetic procedure is done because of societal standard of beauty	
Strongly agree	
Agree	
Neutral	
Disagree	
Strongly disagree	
The cosmetic procedure is performed due to pressure from relatives and friends	
Strongly agree	
Agree	
Neutral	
Disagree	
Strongly disagree	
The cosmetic procedure is done as a means to obtain a job or job requirements	
Strongly agree	
Agree	
Neutral	
Disagree	
Strongly disagree	
The cosmetic procedure is performed due to health problems	
Strongly agree	
Agree	
Neutral	
Disagree	
Strongly disagree	
Cosmetic procedures are performed to increase self-confidence	
Strongly agree	
Agree	
Neutral	
Disagree	
Strongly disagree	
General opinion on aesthetic procedures	
Strongly agree	
Agree	
Neutral	
Disagree	
Strongly disagree	
